# Research note: Genetically diverse avian hepatitis E virus identified in chickens with hepatitis-splenomegaly syndrome in Guangdong Province, China

**DOI:** 10.1016/j.psj.2024.104557

**Published:** 2024-11-22

**Authors:** Xue-Lian Zhang, Yu-Qian Zhang, Chun-Hong Liu, Yan Ma, Shu-Jian Huang, Jian-Wei Shao

**Affiliations:** a School of Animal Science and Technology, Foshan University, Foshan, Guangdong Province, China; b Animal Disease Prevention and Control Center of Guoluo Prefecture, Qinghai Province, China

**Keywords:** Avian hepatitis E virus, Genetic diversity, Novel subtype, Recombination, Hepatitis-splenomegaly syndrome

## Abstract

Avian hepatitis E virus (HEV) is recognized as the primary causative agent of big liver and spleen disease, hepatitis-splenomegaly syndrome, and hepatic rupture hemorrhage syndrome, resulting in substantial economic losses within the global poultry industry. Since its discovery in 1991, diverse strains of avian HEV have been extensively identified worldwide. Epidemiological investigations have demonstrated the wide distribution and genetic diversity of avian HEV strains in China. In this study, avian HEV strains were identified in chickens exhibiting hepatitis-splenomegaly syndrome in Guangdong province, China. Sequence analysis indicated that these strains shared the highest nucleotide sequence identities with genotype 3 strains, and phylogenetic analysis demonstrated that they clustered with strains belonging to avian HEV genotype 3. Moreover, a statistically supported recombination event was detected within one of the avian HEV strain identified in this study. These findings significantly enhance our understanding of the genetic diversity and evolution of avian HEV in chickens, providing new insights for disease prevention and control strategies.

## Introduction

The avian hepatitis E virus (HEV) is the primary causative agent of several diseases in poultry, including big liver and spleen disease (BLS), hepatitis-splenomegaly syndrome (HSS), and hepatic rupture hemorrhage syndrome (HRHS). Other diseases associated with avian HEV are characterized by the accumulation of bloody fluid in the abdomen, vasculitis, amyloidosis in the liver, splenomegaly, and increased mortality (Yugo et al., 2016). Despite the significant economic impact on the global poultry industry, no effective vaccine has been commercialized for avian HEV infection.

Avian HEV is a small, single-stranded positive-sense RNA virus belonging to the family *Hepeviridae*, subfamilies *Orthohepevirinae*, and genus *Avihepevirus* (Purdy et al., 2022). Since its initial identification and sequencing in the United States, avian HEV has been reported in various countries around the world (Yugo et al., 2016). The diverse strains of avian HEV found in chickens can be primarily classified into four genotypes based on full or nearly complete genomes: genotype 1 from Australia and Korea, genotype 2 detected in the USA, Korea, and Spain, and genotypes 3 and 4 reported in Europe, China, and Hungary (Sun et al., 2019; Yugo et al., 2016).

In China, avian HEV infection was confirmed through serological investigations, with the first complete avian HEV genome sequenced from broiler chickens in 2010 (Zhao et al., 2010). Since then, diversified avian HEV strains have been identified across various regions of China (Su et al., 2020). Notably, a novel avian HEV strain, potentially genotype 5, emerged and spread across many Chinese provinces (Su et al., 2020), indicating the significant genetic diversity among avian HEV strains circulating in Chinese chickens, and also highlighting continuous epidemiology investigations are crucial for understanding the dynamics of avian HEV. In this study, genetically diverse avian HEV strains were identified in the livers of chickens with hepatitis-splenomegaly syndrome collected from Meizhou, Zhaoqing, and Yunfu in Guangdong province, China. Furthermore, complete genome sequences were obtained, and the phylogenetic relationship as well as potential recombination events were analyzed.

## Materials and methods

### Sample collection and processing

During November to December 2022, 42 deceased broiler chickens exhibiting hepatitis-splenomegaly syndrome were collected from Meizhou, Zhaoqing, and Yunfu in Guangdong province, China. The chickens were dissected, and liver samples were collected. Postmortem lesions observed in the affected chickens were documented.

Approximately 50 mg of liver tissue was homogenized with 500 μL sterile phosphate-buffered saline (PBS, pH=7.02, GIBCO). Total RNA and DNA were extracted from 200 μL of the homogenates using the Total DNA/RNA Isolation Kit from Omega (Bio-Tek, Norcross, GA), following to the manufacturer's instructions. The extracted RNA and DNA samples were then stored at −70°C for further virus detection.

### Virus detection and complete genome amplification of avian HEV

Avian HEV detection was conducted using nested RT-PCR, following previously established methods (Su et al., 2019). Additionally, the presence of other viral agents in chickens, such as avian leukosis virus (ALV), avian influenza virus (AIV), reticuloendotheliosis virus (REV), chicken infectious anemia virus (CIAV), and fowl adenovirus (FAdV), was detected using published protocols (Li et al., 2020).

After identifying nucleotide sequence similarity and phylogenetic relationships among the screened sequences, positive samples were selected for amplifying the complete genome sequence to better characterize the genetic diversity of avian HEV identified in this study. All PCR products amplified with each primer set were purified using a Gel Extraction kit (TaKaRa, Dalian, China), then cloned into pMD19-T vector (TaKaRa, China), and transformed into *E. coli* competent cells. Positive inserts were determined by PCR, and three selected positive clones were sent for sequencing at Sangon Biotechnology Company (Shanghai, China). To prevent contamination, the PCR mix and template DNA were prepared in a separate room, and aerosol-free pipette tips were used at each stage.

### Sequence comparison and phylogenetic analysis

The final complete genome sequence was assembled and manually edited using SeqMan program (DNASTAR, Madison, WI). Potential open reading frames (ORFs) were annotated by comparing them against the non-redundant protein database. For multiple sequence alignment, the Clustal W program (BioEdit version 7.0) was used, and sequence identity comparisons were performed using MegAlign software (DNASTAR, Madison, WI).

To determine the phylogenetic relationship between the newly identified HEV and other known HEV strains, nucleotide sequences of the complete genome, ORF1, and ORF2 were aligned using the E-INS-i algorithm implemented in the MAFFT program. Maximum-likelihood (ML) trees were reconstructed under the General Time Reversible (GTR) nucleotide substitution model, incorporating a gamma (Γ)-distribution model of among-site rate variation and a proportion of invariable sites (i.e. GTR+Γ+I). Bootstrap support values were calculated from 1,000 replicates using PhyML v3.0. All phylogenetic trees were mid-point rooted for clarity purposes only.

### Recombination analysis

Potential recombination events in the evolutionary history of avian HEV were screened using seven detection methods (RDP, GENECONV, BootScan, MaxChi, Chimera, SiScan, and 3Seq) within Recombination Detection Program, version 4 (RDP4), with default settings. Putative recombination events were identified using a Bonferroni corrected *P* value cutoff of 0.05, and only sequences with significant evidence (*P* < 0.05) of recombination, as detected by three or more methods and confirmed by phylogenetic analysis, were considered to have strong evidence for recombination. Furthermore, the recombinant and parent strains were determined using similarity plot analysis in SimPlot Version 3.5.1.

### Ethics statement

The authors confirm that the ethical policies of the journal, as noted on the journal's author guidelines page, have been adhered to and the appropriate ethical review committee approval has been received. The procedures for sampling and sample processing were approved by the ethics committee of Foshan University. All animals were treated in strict accordance with the Rules for the Implementation of Laboratory Animal Medicine (1998) from the Ministry of Health, China.

## Results and discussion

In this study, 42 deceased broiler chickens exhibiting hepatitis-splenomegaly syndrome were collected, and postmortem lesions analysis showed that the primary pathological changes were mainly observed in the liver and spleen. The key findings included liver hemorrhage, swelling, and even liver rupture in severe cases. Additionally, splenomegaly and splenic hemorrhage were evident. Using a nested RT-PCR assay, all 42 RNA samples tested positive for avian HEV. Subsequently, the PCR products were sequenced, and BLAST analysis revealed that all the sequences shared up to 93.8% nucleotide sequence identity with known avian HEV strains from the GenBank database. Furthermore, tests for other avian viruses, including ALV, AIV, REV, CIAV, and FAdV, were negative in these samples.

To better characterize the genetic diversity of avian HEV strains detected in this study, 10 out of the 42 positive samples were selected for complete genome sequencing based on similarity and phylogenetic analyses of the screening-derived sequences. All sequences determined in this study have been deposited in GenBank under the accession numbers PQ118976-PQ118985. The genomes of these virus strains ranged from 6,633 to 6,655 nucleotides. Sequence comparison analysis revealed they shared 93.1% to 99.6% genome-wide pairwise identity with each other. Furthermore, they exhibited 79.7% to 95.1% nucleotide sequence identity with representative complete genome sequences retrieved from GenBank, with the highest genome-wide nucleotide sequence identity found among sequences belonging to genotype 3. In addition, multiple sequence comparisons based on individual ORFs showed that the complete ORF1 shared nucleotide sequence identities of 79.2% to 94.9% and amino acid sequence identities of 85.8% to 99.6% with these representative strains. The nucleotide sequence identity between the complete ORF2 of avian HEV strains identified herein and the representative strains varied from 80.8% to 95.5%, while the amino acid sequence identity ranged from 89.4% to 99.3%. Additionally, ORF3 between the avian HEV strains identified herein and the representative strains displayed nucleotide sequence identities of 83.5% to 99.2% and amino acid sequence identities of 84.1% to 100.0%.

**Fig. 1:**
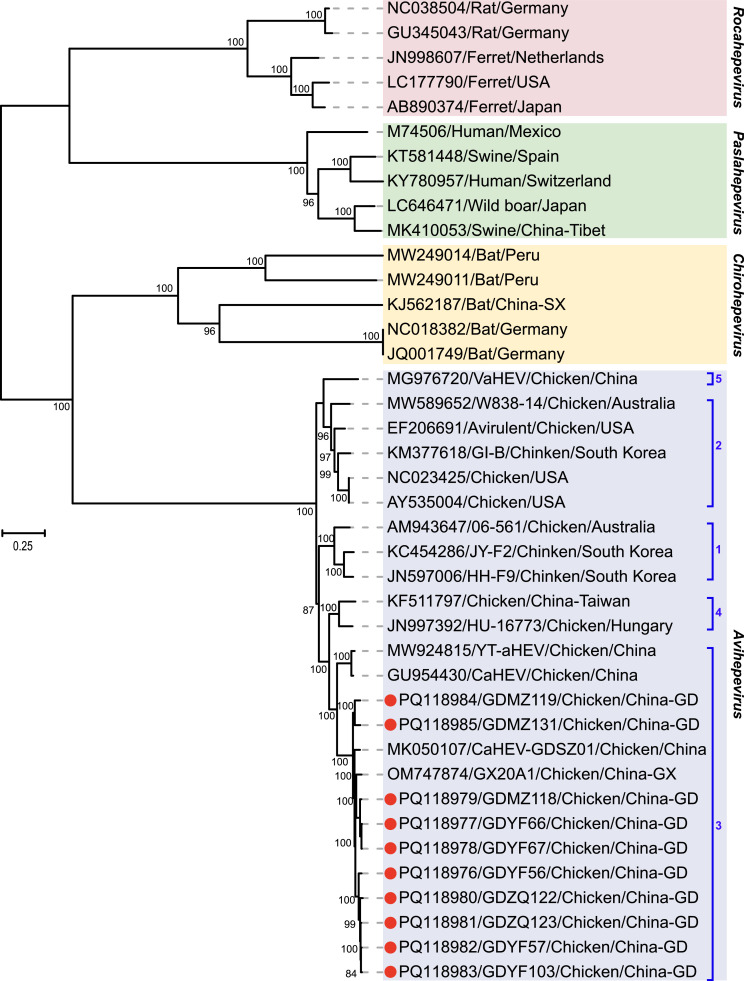
Phylogenetic analysis based on the complete genome sequence of avian HEV and reference HEV isolates. Phylogenetic tree constructed using the maximum likelihood (ML) method in PhyML v3.0, under the GTR+Γ+I model. The analysis includes 1000 replicates of the alignment, with bootstrap values indicated at nodes (>70% shown). The red dot denotes the sequence determined in this study.

**Fig. 2:**
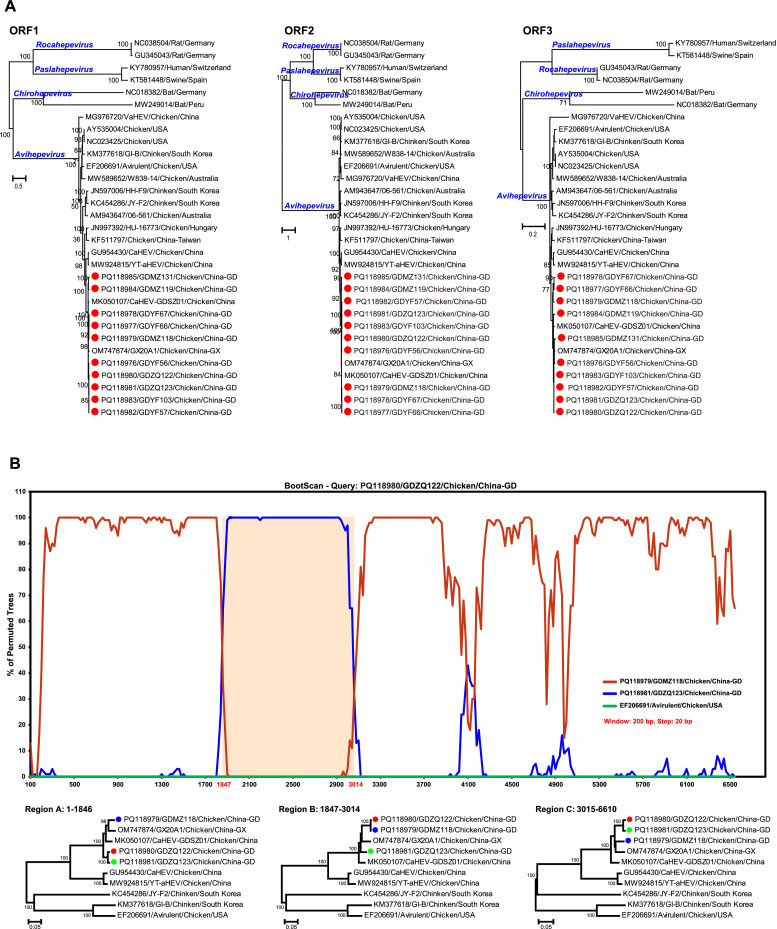
Genetic characterization of avian HEV. (A) Phylogenetic analysis based on the complete nucleotide sequences of ORF1, ORF2, and ORF3 of avian HEV and reference HEV isolates. Phylogenetic tree constructed using the maximum likelihood (ML) method in PhyML v3.0, under the GTR+Γ+I model. The analysis includes 1000 replicates of the alignment, with bootstrap values indicated at nodes (>70% shown). The red dot denotes the sequence determined in this study. (B) Recombination within the genome of avian HEV strain GDZQ122. Bootscan analysis of the complete genome sequence of avian HEV strain GDZQ122 was conducted using SimPlot program. Parameters: 200 bp window size, 20 bp step size, GapStrip: On, 100 replicates, Kimura (2-parameter), and a T/t ratio of 2.0 in Neighbor-Joining analysis. The phylogenetic trees reconstructed based on three regions of the genome (1–1846, 1847–3014, and 3015–6610) using the maximum likelihood (ML) method in PhyML v3.0 under the best-fit substitution model.

The maximum-likelihood phylogenetic tree reconstructed using complete genome sequence revealed that all avian HEV strains identified in this study clustered within the *Avihepevirus* genus, specifically the species *Avihepevirus magniiecur*. These strains grouped with reference strains isolated from chickens and were associated with genotype 3 strains (Fig. 1). Within the genotype 3 subclade, all 10 avian HEV strains identified in this study clustered together with two specific avian HEV strains, CaHEV-GDSZ01 and GX20A1. They exhibited the closest phylogenetic relationship with the avian HEV strain GX20A1, which was isolated from chickens in Jiangxi province, China. Similar phylogenetic relationships were observed in maximum-likelihood trees reconstructed based on the complete nucleotide sequence of ORF1, ORF2, and ORF3 (Fig. 2A). Collectively, these findings, along with previous research, highlight the extensive genetic diversity of avian HEV strains circulating among chicken populations in China. Continuous monitoring of avian HEV infection is essential for effective management of this significant disease in poultry farms.

Recombination is widely recognized as a key driver of viral diversity, crucial for the continuous evolution of viruses and the emergence of new virus species (Simon-Loriere and Holmes 2011). While recombination is relatively common among RNA viruses (Simon-Loriere and Holmes 2011), instances of recombination in HEVs are rarely documented (He et al., 2023). To our knowledge, only one study has reported potential recombination event in avian HEV (Asif et al., 2021). To investigate potential recombination events in the evolution of avian HEV, we analyzed 26 complete genomes retrieved from GenBank, alongside 10 sequences obtained in this study. Multiple methods within the RDP program identified a statistically significant recombination event in the avian HEV strain GDZQ122. Simplot analysis using the bootscan method confirmed this recombination event and indicated two recombination breakpoints at nucleotide positions 1,847 and 3,014 (Fig. 2B). Consequently, the genome of GDZQ122 can be divided into three regions with distinct evolutionary histories: region A (nucleotides 1–1846), region B (1847–3014), and region C (3015–6610). In regions A and C, GDZQ122 was most closely related to GDMZ118, while in region B, it showed a closer relationship to GDZQ123. Phylogenetic analyses of these regions revealed significant topological changes in strain GDZQ122, providing strong evidence for this recombination event (Fig. 2B). Notably, the potential parental sequences were identified as avian HEV strains GDZQ123 and GDMZ118, which were collected from Zhaoqing and Maoming in Guangdong province. The recombination event observed between strains from these different regions suggests potential genomic exchange of the virus across diverse locations. It should also be noted that the recombination analysis was based on a limited number of avian HEV complete genome sequences. As more avian HEV sequences are deposited in GenBank, the role of recombination in the evolutionary dynamics of avian HEV will become clearer.

## Disclosures

The authors declare that the research was conducted in the absence of any commercial or financial relationships that could be construed as a potential conflict of interest.

## Declaration of competing interest

The authors declare that they have no known competing financial interests or personal relationships that could have appeared to influence the work reported in this paper.
